# The German Alliance Against Depression and suicide rates: A retrospective analysis

**DOI:** 10.1371/journal.pone.0254133

**Published:** 2021-07-01

**Authors:** Judith Köhler, Ines Heinz, Roland Mergl, Anne Elsner, Ulrich Hegerl

**Affiliations:** 1 Department of Psychiatry and Psychotherapy, Medical Faculty, University Leipzig, Leipzig, Germany; 2 German Alliance Against Depression, Leipzig, Germany; 3 Universität der Bundeswehr München, Institute of Psychology, Neubiberg, Germany; 4 German Depression Foundation, Leipzig, Germany; 5 Department of Psychiatry, Psychosomatic Medicine and Psychotherapy, University Hospital Frankfurt, Goethe University Frankfurt (Distinguished Professorship funded by Dr. Senckenbergische Stiftung), Frankfurt am Main, Germany; University of Toronto, CANADA

## Abstract

Supported by the German Alliance Against Depression, 82 regions in Germany launched their own community-based multi-level intervention programs targeting both depression and suicidal behavior prior to January 2016. Sixteen of these regions have implemented the full 4-level intervention program comprising 1) training of General Practitioners, 2) a public awareness campaign, 3) training of community facilitators and 4) support for depressed patients and their relatives for at least three years. The aim of the study was to examine possible suicide prevention effects in these sixteen 4-level intervention regions (comprising a population of 6,976,309) by 1) comparing the annual suicide rates during the 3-year intervention period to a 10-year baseline and 2) comparing these differences to corresponding trends in Germany after excluding all intervention regions (Germany-IR). Primary outcome was the annual rate of suicides. Analyses included negative binomial regression models. When examining differences between suicide rates during the intervention period compared to the baseline period, only a trend towards a significant reduction was found. This reduction of suicides in the sixteen 4-level intervention regions did not differ from that in Germany-IR as control. The interpretation of these findings has to take into account that the training of General Practitioners, police and other community facilitators might have improved the recognition of suicides, thus increasing detection rates. Furthermore, destigmatizing effects of the public awareness campaigns might have increased the number of suicides by lowering suicide threshold (“normalization”) for those at risk and by decreasing the rate of suicides deliberately hidden by suicide victims or their relatives.

## Introduction

Suicide remains a leading cause of death worldwide and suicide prevention has been recognized as a major public health objective [1].

Multifaceted community-based interventions such as the 4-level intervention program developed and promoted by the German Alliance Against Depression (GAAD) and the European Alliance Against Depression (EAAD) [2] are considered a promising approach to prevent suicidal behavior. This 4-level intervention program has been named as a successful example in the context of suicide prevention by the World Health Organization and the European Commission [1, 3]. The program simultaneously targets the following two partly overlapping aims: Improving care for individuals with depression and preventing suicidal behavior. Combining the two aims is based on both the well-documented large therapeutic deficits concerning depression [4] and the important role depression plays as a risk factor for suicidal behavior [1, 5]. The community-based 4-level intervention program comprises simultaneous activities at four levels: 1) training of General Practitioners (GPs), 2) a public awareness campaign, 3) training of community facilitators and 4) support for patients and their relatives [2].

So far, the GAAD has supported over 80 regions in Germany with implementing their own suicide prevention interventions. Supported by the EAAD, the 4-level intervention program was also implemented in several European countries as well as overseas (Canada, Australia and Chile).

Several prospective studies provide evidence that the 4-level intervention program is associated with reductions in suicides or in suicidal acts (suicides + attempted suicides) [6–8]. Significant effects on suicidal acts in intervention regions compared to control regions were found in both the pilot region of Nuremberg (Germany) [6] and in a region of Portugal (Amadora) within the research project OSPI-Europe (“Optimizing Suicide Prevention Programs and their Implementation in Europe”) [9]. Because of the low base rate of suicides, in these studies suicidal acts were used as primary outcome in order to improve statistical power [9]. Two other studies from Germany (Regensburg) [7] and Hungary (Szolnok) [8], for which no data on attempted suicides were available, found significant effects on completed suicides. In Szolnok, suicide rates significantly decreased during the 4-level intervention compared to both a control region (Szeged) and the country overall. In Regensburg, suicide rates significantly decreased compared to a 5-year baseline period but not compared to the trend in Germany. However, within the research project OSPI-Europe no significant effect of the 4-level intervention on suicidal acts was observed in other regions of Germany (Leipzig), Hungary (Miskolc) and Ireland (Limerick) [9].

A small number of other multilevel community-based suicide prevention programs, which did not follow the 4-level intervention program promoted by EAAD, also appeared to have preventive effects on suicides [10, 11], while others did not [12]. Given the complexity of these community-based interventions as well as a manifold of uncontrollable and even unforeseeable intervening factors (for process and implementation research concerning the 4-level intervention program see Harris et al. [13, 14]), it is not surprising that positive effects on suicidal behavior were not found consistently across all studies.

The aim of the present retrospective study was to examine a possible suicide prevention effect in those sixteen regions that had implemented the full 4-level intervention program for at least three years by addressing two questions:

1. Did the annual suicide rates decrease during the three years after the start of the 4-level intervention (intervention period) compared to a 10-year baseline?
2. Is this decrease more pronounced than that observed for the same time period in Germany after excluding regions that were using the intervention program (Germany-IR)?

## Methods

### Sample

Since the introduction of the first Alliance Against Depression in Nuremberg in 2001 [6], 82 regional alliances have joined the GAAD prior to January 2016. This nationwide expansion comprises a heterogeneous mixture of major cities and rural areas. The GAAD supports the regional alliances with intervention and evaluation materials, provides training within the framework of trainer programs and continuous guidance. However, regional alliances must look for local sponsors and volunteers in order to run their interventions. Correspondingly, the regional alliances differ greatly in terms of the intensity of the interventions, depending on the resources available and the stakeholders involved. For the current study, all 82 Alliances Against Depression, launched prior to January 2016, were contacted and asked for information within the context of a survey about their activities at each of the four intervention levels, e.g. the number of trained GPs and community facilitators or printed flyers and posters. Based on this information, sixteen 4-level intervention regions, comprising a population of 6,976,309, met the following inclusion criteria: Activities at all four intervention levels had been carried out for at least three years, data on suicides were available for that specific region (provided by the Statistical State Offices of Germany [15]) and activities had started prior to the year 2012. 60 regions started their interventions prior to the year 2012. The inclusion of regions starting their 3-year intervention after 2012 would have been problematic, since after 2014, the concept of confidentiality regarding the reporting of suicides was modified in some federal states of Germany in order to impede the identification of single suicide cases [Thomas Graf on behalf of the Statistical State Office of Germany, personal communication, September 2019]. This introduced a systematic bias as suicides were shifted to the category ‘undetermined deaths’ for confidentiality reasons in smaller regions.

Of the 82 regional Alliances Against Depression, 44 regions were excluded because they had not implemented all four levels, 14 were excluded for not having implemented their activities for at least three years, and one region was excluded due to unavailability of suicide mortality data. Two regions were excluded, because they were districts in large cities (Berlin, Hamburg) and it was difficult to detect changes in suicide rates for individual districts. Five regions had joined the GAAD, but never started any activities due to different reasons, leaving a total of sixteen intervention regions running the full 4-level intervention program for the present analyses.

In order to compare changes in suicide rates of the sixteen intervention regions with the national trend, suicide rates for Germany were calculated. For this calculation, regions in which any GAAD-intervention had taken place were excluded, resulting in Germany-IR as the control area. Thus, Germany-IR comprised neither the sixteen 4-level intervention regions mentioned above, nor the other 51 regions, which had started at least some intervention activities, but did not meet the inclusion criteria. The annual number of suicides and population data for the 67 regions with any GAAD activity at any time were subtracted from the overall suicide and population numbers in Germany. The annual number of suicides and population data of control and intervention regions for the period of 1991 to 2014 were obtained from the Statistical State Offices of Germany [16]. This data is officially recorded, fully anonymized and publicly accessible. A review by an ethics committee was therefore not necessary.

### Intervention

The GAAD 4-level intervention program comprises simultaneously implemented activities at four levels to improve care of individuals suffering from depression and to prevent suicidal behavior. The program is manualized and provides intervention regions with step-by-step guidance on how to implement activities within each of the four levels as well as information on how to coordinate their simultaneous implementation. At Level 1, GPs are trained in recognizing and treating depression and provided with materials such as patient screening questionnaires and educational brochures. At Level 2, the aim is to raise awareness of depression as a treatable illness that can affect everyone in the general public via mass media and public relations campaigns. At Level 3, community facilitator trainings are conducted for various stakeholders including clergy, police, teachers and social workers. Level 3 also includes reaching out to and working with journalists in order to avoid the ‘Werther effect’ [17, 18] by increasing adherence to recommended media guidelines for the reporting of suicide. Finally, Level 4 provides support for patients and their relatives through self-help groups and leisure activities, advertisements for crisis lines, especially for high-risk groups, and online forums for patients with depression. Additional intervention elements that aim at reducing access to lethal means include the identification and securing of possible suicide hotspots in the intervention region or raising awareness among GPs concerning the package size of psychopharmacological drugs with high risk of overdose. Local alliances decide on the intensity of their intervention activities depending on their resources, since GAAD activities are mainly based on voluntary engagement and do not receive any official governmental funding.

### Suicide mortality data

The primary outcome variable was the annual crude rate of suicides in Germany from the Statistical State Office of Germany [16]. Suicide was defined as intentional self-harm resulting in death (International Classification of Diseases, 10^th^ revision [19], categories X60-X84 from 1998 to 2014). Suicide mortality data from 1991 to 1997 were based on the ICD-9 intentional self-harm codes E950-E959 [20]. Suicide rates were operationalized as crude rates by calculating the number of suicides per 100,000 inhabitants. Annual suicide rates were also stratified by gender (male vs. female).

### Effect size calculation

In order to determine the minimal change in the annual frequency of suicides within the 4-level intervention regions necessary to result in significant corresponding differences between the sixteen intervention regions and Germany-IR, we performed an effect size calculation based on Fisher’s exact test using the software G*Power (Version 3.1.9.6. [21]). When considering mean suicide numbers in the baseline period (4-level intervention regions: 941; Germany-IR: 7856) and a decrease of suicides of -10.8% in Germany-IR, a decrease of suicides of at least -27% in the 4-level intervention regions would be required, assuming a statistical power of 0.80 and a significance level of alpha = 0.05 (two-tailed testing). This corresponds to a delta of at least 16.2%.

### Data analysis

Regarding research question 1, means of the annual suicide rates for the 10-year baseline period and for the 3-year intervention period were compared for the sixteen regions, which had implemented the 4-level intervention (intervention regions).

The intervention period comprised the 3-year period after the initiation of the intervention. A baseline period of ten years was chosen in order to account for any normal fluctuations in the annual suicide rate. Since our outcome variables were zero-inflated, negative binomial regression models were used. Incidence ratios for the annual suicide rates between the intervention period and the baseline period with corresponding 95% confidence intervals (95% CI) were computed for the intervention regions and Germany-IR. The offset term ‘log(population number per year)’ was used to adjust for any annual changes in the population. Mann-Whitney U tests were used to compare intervention periods with baseline periods. Next, the magnitude and direction of the percentage change (PC) in annual suicide rates were calculated for each region. The percentage change was derived from the incidence ratio (IR) according to the formula: PC = (IR-1) * 100. The mean percentage change was compared with 0 (reflecting no difference between the intervention and baseline period) by using a one-sample t test. In this context, a weighting factor for the mean population size of the intervention regions in a 14-year period (10-year baseline period + year in which the local Alliance Against Depression had been founded + 3-year intervention period) was used.

The incidence ratio of the annual number of completed suicides in the 3-year intervention period for Germany-IR was compared to the corresponding numbers in the 10-year baseline period for Germany-IR, based on a negative binomial regression analysis with log(annual population number) as offset term. In a next step, the mean corresponding incidence ratio was compared with 1 (reflecting no difference between the intervention and baseline time interval) by using a one-sample t test. Unweighted results were given. For subsequent analyses, ten time periods were defined depending on the various implementation years of the intervention regions. For example, one region began implementing the GAAD program in 2001 and therefore had a baseline time interval of 1991–2000 and an intervention time interval of 2002–2004.

Next, research question 2 was considered: The difference between the percentage change in a specific intervention region and the percentage change in Germany-IR (ΔPC = PCintervention–PCGermany-IR) was computed. An effect in the expected direction was given for negative values of ΔPC. A one-sample t test was chosen to determine whether ΔPC was significantly different from 0. In this context, the above-mentioned weighting factor for the mean population size of the intervention regions was used. ΔPC was normally distributed, as revealed by Kolmogorov-Smirnov tests (*p* > 0.05).

## Results

**Fig 1:**
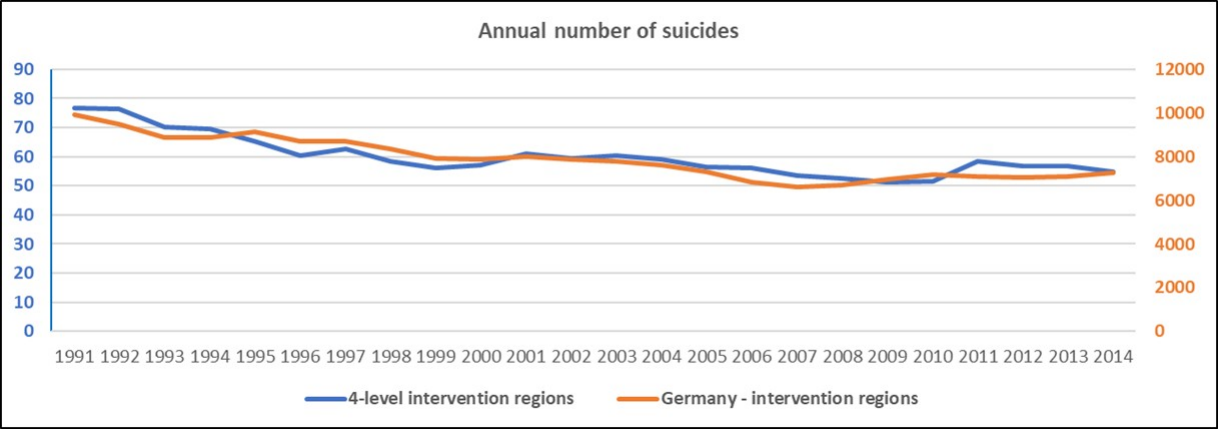
Annual number of suicides in 4-level intervention regions and in Germany-IR.

The mean annual number of suicides for the sixteen 4-level intervention regions and Germany-IR in the 10-year baseline and the 3-year intervention period are summarized in Table 1, for the corresponding population numbers see S1 Table. The time course of the number of suicides in the period 1991–2014 has been illustrated in Fig 1.

**Table 1:** Mean annual number of suicides in the baseline and intervention period.

Variables	Men and women	Men	Women
**4-level intervention regions**			
**Mean number of suicides per year in the baseline period (SD)**	940.90 (30.13)	667.10 (24.68)	273.80 (16.77)
**Mean number of suicides per year in the intervention period (SD)**	859.00 (37.99)	610.00 (47.70)	249.00 (14.42)
**Germany-IR**^**a**^			
**Mean number of suicides per year in the baseline period (SD)**	7856.22 (423.16)	5821.96 (269.38)	2034.26 (154.08)
**Mean number of suicides per year in the intervention period (SD)**	7006.79 (158.96)	5284.65 (111.71)	1722.15 (55.18)

SD: standard deviation; Germany-IR: Germany without intervention regions.

^a^ The weighted arithmetical means for the number of suicides per year are given. The weights refer to the year in which the German Alliances Against Depression was founded.

Changes regarding suicide rates in intervention regions as well as Germany-IR are summarized in Table 2.

**Table 2:** Changes in suicide rates in 4-level intervention regions and in Germany-IR.

Variables	Men and women	Men	Women
**Percentage change (4-level intervention regions)**^**a**^	-7.63% (p = 0.072^+^)	-7.57% (p = 0.087^+^)	-8.02% (p = 0.120)
**Percentage change (Germany-IR)**^**b**^	-10.05% (p < 0.001***)	-8.6% (p < 0.001***)	-14.4% (p < 0.001***)
**Percentage change (4-level intervention regions)–Percentage change** **(Germany-IR) (ΔPC)**^**c**^	+1.81% (p = 0.640)	+0.38% (p = 0.93)	+5.93% (p = 0.189)
**Effect (ΔIR/ΔPC) in the expected direction**^**d**^	No	no	no

IR: incidence ratio; PC: percentage change; vs.: versus;

^a^ Percentage changes were computed according to the formula PC = (IR-1)*100.

^b^ Percentage changes were computed according to the formula PC = (IR-1)*100. Unweighted results are presented; thus, the mean delta percentage change (see below) cannot be derived from the (weighted) percentage change values presented in the upper line.

^c^ The mean delta percentage change (ΔPC) was compared with 0. In this context, a weighting factor for the mean population size of the intervention regions in a 14-year period (10-year baseline period + year in which the local Alliance Against Depression was founded + 3-year intervention period) was used. The given p values refer to this test.

^d^ An effect in the expected direction was given for negative values of ΔPC.

^+^p<0.10; * p<0.05; ** p<0.01;

*** p<0.001.

See S2 Table for the incidence ratios for the 44 excluded intervention regions which had not implemented all four levels and information on the levels where interventions took place.

Regarding the 4-level intervention regions (*n* = 16), a statistical trend for lower suicide rates compared to a 10-year baseline period was found (for percentage changes based on incidence ratios, see Table 2). The mean percentage change in annual suicide rates for these regions was -7.63% (SD = 15.78%; range: -39.70% to +32.40%). However, this value was not significantly different from 0 (mean difference = -7.63%; 95% CI: -16.04% to +0.78%; t = -1.93; df = 15; p = 0.072).

The mean delta percentage change (ΔPC) based on the corresponding delta incidence ratios for the sixteen intervention regions in Germany, which had implemented all four intervention levels for at least three consecutive years was +1.81% (SD = 15.19%; range: -28.80% to +35.10%). This difference was not significantly different from 0 (mean difference = +1.81%; 95% CI: -6.28% to +9.90%; *t*(15) = 0.48; *p* = 0.640).

When stratified by gender, there was no significant reduction in suicide rates in the intervention regions during the intervention period compared to the baseline period (men: -7.57%, women: -8.02%). There was also no gender-specific difference between the intervention regions and Germany-IR in changes of suicide rates.

## Discussion

The aim of this study was to examine a possible suicide prevention effect in the 4-level intervention regions. The results show a trend towards a reduction in suicide rates in the sixteen 4-level intervention regions during the 3-year intervention period compared to the 10-year baseline period. However, this reduction was not more pronounced than corresponding changes in Germany-IR, resulting in a positive mean delta percentage change. This difference was not significantly different from 0. Also, when stratifying suicide rates by gender, no difference was found.

This lack of evidence concerning a reduction in suicide rates is contrary to previous findings for the German city of Regensburg [7], which showed a significant reduction of suicide rates compared to baseline, and for the Hungarian city of Szolnok [8], which showed a significant reduction of suicide deaths compared to national suicide trends and a control region.

When interpreting our results, the following aspects have to be taken into account.

A recognition bias, explaining the negative findings, cannot be excluded. Raising awareness in GPs, police and other community facilitators concerning suicide through the work of regional alliances might have led to a higher sensitivity and subsequent increase of the number of detected suicides in intervention regions. This bias was already considered to play a role within the pilot study of the Alliance Against Depression of Nuremberg [6]. In Nuremburg during the two intervention years, the number of suicidal acts decreased by 24% in the intervention region, compared to the baseline year and to the control region. In this pilot study, the significant decrease of suicidal acts in the intervention region compared to both baseline and the control region was more pronounced for high-risk methods (e.g., jumping, hanging). The probability of being detected as a suicide is higher for these methods than it is for low-risk methods such as intoxication [6]. Furthermore, destigmatizing suicidal behavior might have reduced the number of suicides deliberately hidden by the suicide victim itself, their family or the doctors. Both factors would introduce a bias inflating the suicide numbers in the intervention regions.

In order to better understand why suicides in the intervention regions didn’t show a significantly more pronounced decline compared to the control region, it would be insightful to compare the annual average percentage change of both suicides and undetermined deaths in the intervention regions and control region. It can be assumed that 9 of 10 undetermined deaths represent hidden suicides [22] and according to a time trend analysis of suicides and undetermined deaths in Germany for the period 1991–2002 [23] a significant decrease of the annual suicide rates correlated with a significant increase of undetermined death rates in the subgroup of the elderly (75 years and older). Analyses taking into account undetermined deaths could help to better comprehend the underlying mechanisms of the 4-level-approach, i.e., whether a higher sensitivity and subsequent increase of the number of detected suicides in intervention regions leads to a corresponding substantial decrease of the number of undetermined deaths. Such analyses would have gone beyond the scope of this manuscript and should be addressed in following studies.

However, we need to consider that our findings reflect a lack of anti-suicidal effects. There are several factors with a possible negative impact on suicidality.

Raising public awareness on suicide through public relation activities may have increased the cognitive availability of suicidal behavior in the population [24]. This factor and destigmatizing suicidal behavior (“normalization” of suicide) might lower the suicide threshold for individuals at risk. Individuals suffering from depression often feel that they are a burden to others and that it would be better if they were no longer alive. The stigma associated with suicide keeps them from committing suicide for the sake of their families. Suicide prevention campaigns targeting the general population can also trigger secondary and unwanted coverage on social media and elsewhere which cannot be controlled concerning wording and possible negative effects on people at risk (e.g. ‘Werther effect’ [17, 18]). The extensive media coverage of German national soccer player Robert Enke’s railway suicide in 2009 was followed not only by an acute but also a long-term increase (two years) in railway suicides [25] and also points to the risks associated with public discussions on suicide [26]. Within the 4-level intervention program, it is recommended to give a strong focus on suicide prevention in trainings for professionals and to shift the focus towards depression when addressing the general public. However, it is unknown to what extent this recommendation was considered by intervention regions and what the secondary reporting on suicidal behavior looked like. Examining these aspects is important for future research.

The intensity of intervention activities in the various intervention regions is another aspect to consider. As the 4-level intervention program and the regional alliances were not part of a national suicide prevention strategy, they vary significantly according to structure, budget and organization. Many coordinators reported difficulties in finding the necessary resources to carry out the campaign with the desired intervention intensity. Detailed data on the intensity of the interventions at the different levels are not available.

Finally, due to the high mobility of the population in everyday life and well-connected communication, the campaign might have spread outside of the intervention regions reducing the chance to find different effects between 4-level intervention regions and Germany-IR.

No data on attempted suicides were available in this study. This limits the conclusions concerning possible effects on suicidal behavior and strongly reduces the statistical power of this analysis. The actual decrease of the number of suicides in the 4-level intervention regions (-8.71%) was clearly below -27%, which would have been required to obtain statistical significance. The delta (4-level intervention regions–Germany-IR) of +2.09% was also below the corresponding cut-off score for statistical significance (16.2%).

Mental disorders are among the most important risk factors for suicides, especially depressive disorders are closely related to suicidal behavior [5, 27]. In addition to depressive disorders, other psychiatric diagnoses or factors such as symptoms of anxiety or metabolic parameters can influence suicidal behavior [28].

Finally, it must also be borne in mind that the restriction of access to lethal means, which was an element at intervention Level 3, is an effective element in suicide prevention programs, but needs time to show possible effects. Once suicide hot spots have been identified, intervention program activities aimed at securing such hotspots (e.g. bridges) require complex political and administrative decisions which often take several years until required measures are taken [9].

The fact that suicide trends in the intervention regions did not differ from that in the control regions could be explained by several methodological problems. This limits the strength of the conclusions which can be drawn from this retrospective study.

Future analyses should focus on the potential problem of the intervention program affecting the recognition of suicidal behavior, possibly leading to a higher detection rate of suicides or a lower rate of deliberately hidden suicides. Another scientific focus should be on unwanted effects of anti-suicidal campaigns due to destigmatizing and normalizing suicide, possibly lowering suicide thresholds. Finally, unfavorable and uncontrollable secondary social media reporting (‘Werther effect’ [17, 18]) and discussions (e.g. discussions about suicidal methods) triggered by anti-suicidal campaigns require further scientific attention.

In summary, further studies are needed to confirm the reported findings.

## Supporting information

S1 Table Mean annual population number in the baseline and intervention period. (DOCX)

S2 Table The mean incidence ratio of the annual number of suicides in the index time interval (intervention period) compared to the 10-year baseline period for 44 German regions with alliances against depression implementing at least one level of the full GAAD program, but not the full program for at least three years. (DOCX)

S1 File Data. (XLSX)
