# Supplementary material for: The German Alliance Against Depression and suicide rates: A retrospective analysis
Source: PLoS One. 2021 Jul 1;16(7):e0254133. doi: 10.1371/journal.pone.0254133 (PMC8248967; doi:10.1371/journal.pone.0254133)
Supplement: S1 Table — (DOCX) [file pone.0254133.s001.docx]

**S1 Table: Mean annual population number in the baseline and intervention period**

| Variables | Men and women | Men | Women |
| --- | --- | --- | --- |
| 4-level intervention regions | | | |
| Mean annual population number in the baseline period (SD) | 6919920 (48680) | 3356682 (29496) | 3563238 (19375) |
| Mean annual population number in the intervention period (SD) | 7030942 (8896) | 3419438 (4612) | 3611504 (4297) |
| Germany-IR^a^ | | | |
| Mean annual population number in the baseline period (SD) | 57507229 (108660) | 28162609 (81385) | 29344620 (37554) |
| Mean annual population number in the intervention period (SD) | 56812342 (622696) | 27881085 (314032) | 28931258 (310173) |

SD: standard deviation; Germany-IR: Germany without intervention regions.

^a^ The weighted arithmetical means for the annual population number are given. The weights refer to the year in which the German Alliances Against Depression was founded.
