# Supplementary material for: The German Alliance Against Depression and suicide rates: A retrospective analysis
Source: PLoS One. 2021 Jul 1;16(7):e0254133. doi: 10.1371/journal.pone.0254133 (PMC8248967; doi:10.1371/journal.pone.0254133)
Supplement: S2 Table — (DOCX) [file pone.0254133.s002.docx]

**S2 Table: The mean incidence ratio of the annual number of suicides in the index time interval (intervention period) compared to the 10-year baseline period for 44 German regions with alliances against depression implementing at least one level of the full GAAD program, but not the full program for at least three years**

| **Region** | **Levels** | | | | | **Incidence ratios** | | | |
| --- | --- | --- | --- | --- | --- | --- | --- | --- | --- |
|  | **1** | **2** | **3** | **4** | **Total number** | **IR^a^** | **Ref. value^b^** | **ΔIR^c^** | **Effect in the expected direction (y/n)^d^** |
| Nürnberg | X | X | X | X | 4 | 0.958 | 0.877** | 0.081 | n |
| Kempten | ? | ? | ? | ? | ? | 1.089 | 0.856** | 0.233 | n |
| Erlangen | - | X | X | X | 3 | 0.975 | 0.856** | 0.119 | n |
| Lübeck | ? | ? | ? | ? | ? | 1.071 | 0.856** | 0.215 | n |
| Schwerin | ? | ? | ? | ? | ? | 0.657 | 0.830** | -0.173 | y |
| Fürth | - | X | - | X | 2 | 0.889 | 0.830** | 0.059 | n |
| Groß-Gerau | X | X | X | X | 4 | 0.955 | 0.830** | 0.125 | n |
| Ingolstadt | ? | ? | ? | ? | ? | 0.983 | 0.830** | 0.153 | n |
| Flensburg | ? | ? | ? | ? | ? | 1.023 | 0.821** | 0.202 | n |
| Aachen | ? | ? | ? | ? | ? | 1.092 | 0.821** | 0.271 | n |
| Würzburg | ? | ? | ? | ? | ? | 1.128 | 0.821** | 0.307 | n |
| Ostfriesland | ? | ? | ? | ? | ? | 0.756 | 0.821** | -0.065 | y |
| Düsseldorf | X | X | X | X | 4 | 0.704 | 0.821** | -0.117 | y |
| Duisburg | X | X | X | X | 4 | 0.687 | 0.821** | -0.134 | y |
| Schleswig | ? | ? | ? | ? | ? | 0.804 | 0.846** | -0.042 | y |
| Memmingen-Unterallgäu | ? | ? | ? | ? | ? | 0.649 | 0.846** | -0.197 | y |
| Dillingen | ? | ? | ? | ? | ? | 0.970 | 0.846** | 0.124 | n |
| Region Düren | ? | ? | ? | ? | ? | 0.968 | 0.846** | 0.122 | n |
| Neckar-Alb (Tübingen) | X | X | X | X | 4 | 1.056 | 0.846** | 0.210 | n |
| Schwarzwald/ Baar/ Heuberg/ Zollern-Alb (Rottweil) | ? | ? | ? | ? | ? | 0.836 | 0.846** | -0.01 | y |
| Stadtroda | ? | ? | ? | ? | ? | 0.662 | 0.846** | -0.184 | y |
| Donau-Bodensee (Sigmaringen) | ? | ? | ? | ? | ? | 1.034 | 0.846** | 0.188 | n |
| Wuppertal | - | X | - | X | 2 | 0.885 | 0.846** | 0.039 | n |
| Dortmund | ? | ? | ? | ? | ? | 0.878 | 0.891* | -0.013 | y |
| Darmstadt | X | X | X | X | 4 | 0.791 | 0.891* | -0.100 | y |
| Kreis Steinfurt | X | X | X | X | 4 | 0.960 | 0.891* | 0.069 | n |
| Essen | - | X | X | X | 3 | 1.052 | 0.935 | 0.117 | n |
| Region Hannover | - | X | X | X | 3 | 0.906 | 0.935 | -0.029 | y |
| Nürnberger Land | X | X | X | X | 4 | 1.091 | 0.935 | 0.156 | n |
| Neckar-Odenwald-Kreis (Mosbach) | - | X | X | X | 3 | 1.028 | 0.935 | 0.093 | n |
| Rhein-Neckar-Süd (Wiesloch) | X | X | X | X | 4 | 0.810* | 0.963 | -0.153 | y |
| Dithmarschen | ? | ? | ? | ? | ? | 0.886 | 0.963 | -0.077 | y |
| Herne | - | X | X | X | 3 | 1.024 | 0.963 | 0.061 | n |
| Bochum | - | X | X | X | 3 | 1.127 | 0.963 | 0.064 | n |
| Mainz | ? | ? | ? | ? | ? | 0.792* | 0.963 | -0.171 | y |
| Münster | - | X | - | X | 2 | 1.084 | 0.973 | 0.111 | n |
| Kreis Recklinghausen | X | X | - | X | 3 | 1.143 | 0.973 | 0.170 | n |
| Bernkastel-Wittlich | ? | ? | ? | ? | ? | 1.028 | 0.973 | 0.055 | n |
| Rhein-Ahr-Wied (Andernach) | - | X | - | - | 1 | 0.893 | 0.973 | -0.080 | y |
| Plauen/ Vogtlandkreis | ? | ? | ? | ? | ? | 1.092 | 0.988 | 0.104 | n |
| Gütersloh | ? | ? | ? | ? | ? | 1.079 | 0.988 | 0.091 | n |
| Landau-Südliche Weinstraße (Klingenmünster) | ? | ? | ? | ? | ? | 0.990 | 0.988 | 0.002 | n |
| Magdeburg und Halle (Saale) | ? | ? | ? | ? | ? | 1.404 | 0.988 | 0.416 | n |
| Rhein-Hunsrück (Boppard) | ? | ? | ? | ? | ? | 0.805 | 0.988 | -0.183 | y |

**Notes:** IR: incidence ratio; n: no; ref.: reference; y: yes.

^a^ The incidence ratio of the annual number of completed suicides in the index time interval (three-year intervention period) compared to the corresponding numbers in the control time interval (ten-year baseline period) (reference) for German regions with local alliances against depression implementing at least one level of the GAAD (German alliance against depression) program, based on a negative binomial regression analysis with log(annual population number) as offset term. ^b^ The incidence ratio of the annual number of completed suicides in the index time interval (three-year intervention period) compared to the corresponding numbers in the control time interval (ten-year baseline period) (reference) for Germany without the corresponding alliances against depression with at least one level of GAAD activities, based on a negative binomial regression analysis with log(annual population number) as offset term. ^c^ ΔIR = IR_intervention region_ – IR_Germany without intervention regions_

^d^ An effect in the expected direction was given for negative values of ΔIR.

*p≤0.05; **p≤0.01; ***p≤0.001 (two tailed testing) (according to results of Mann-Whitney U tests for the comparison of intervention and baseline periods)

The total number of levels refers to the total number of levels at which activities for suicide prevention had taken place.

Level 1: Cooperation with primary care; level 2: Awareness campaign; level 3: Education of community facilitators; level 4: Services for patients, relatives and high-risk groups to target depression and suicidal behavior.
